# Recommendations for resuming elective hip and knee arthroplasty in the setting of the SARS-CoV-2 pandemic: the European Hip Society and European Knee Associates Survey of Members

**DOI:** 10.1007/s00167-020-06212-0

**Published:** 2020-08-18

**Authors:** N. P. Kort, E. Gómez Barrena, M. Bédard, S. Donell, J.-A. Epinette, B. Gomberg, M. T. Hirschmann, P. Indelli, Ismail Khosravi, T. Karachalios, M. C. Liebensteiner, B. Stuyts, R. Tandogan, B. Violante, L. Zagra, M. Thaler

**Affiliations:** 1 CortoClinics, Schijndel, The Netherlands; 2 Dept of Orthopaedic Surgery and Traumatology, Hospital La Paz, Universidad Autónoma de Madrid, Madrid, Spain; 3 Département de Chirurgie Orthopédique, CHU de Québec-Université Laval, Quebec City, QC Canada; 4 Norwich Medical School, University of East Anglia, Norwich, UK; 5 Center for Research and Documentation in Arthroplasty, Lille, France; 6 OA Centers for Orthopaedics, Portland, ME USA; 7 Department of Orthopaedic Surgery and Traumatology, Kantonsspital Baselland, (Bruderholz, Liestal, Laufen), 4101 Bruderholz, Switzerland; 8 University of Basel, Basel, Switzerland; 9 Department of Orthopaedic Surgery, Stanford University School of Medicine, Stanford, CA USA; 10 International Committee American Academy Hip and Knee Surgeons (AAHKS), Rosemont, IL USA; 11 Department of Orthopaedic Surgery, Medical University of Innsbruck, Anichstrasse 35, 6020 Innsbruck, Austria; 12 Orthopaedic Department, School of Health Sciences, Faculty of Medicine, University General Hospital of Larissa, University of Thessalia, Thessalia, Greece; 13 Department of Orthopedic Surgery and Traumatology, GZA Hospitals, Antwerp, Belgium; 14 Ortoklinik and Cankaya Orthopedics, Ankara, Turkey; 15 Orthopaedic Department, Istituto Clinico Sant’Ambrogio IRCCS Galeazzi, Milan, Italy; 16 Hip Department IRCCS Istituto Ortopedico Galeazzi, Milan, Italy

**Keywords:** COVID-19, Primary arthroplasty, Hip, Knee, Recommendations, Survey

## Abstract

**Purpose:**

The COVID-19 pandemic has disrupted the health care system around the entire globe. A consensus is needed about resuming total hip and knee procedures. The European Hip Society (EHS) and the European Knee Association (EKA) formed a panel of experts that have produced a consensus statement on how the safe re-introduction of elective hip and knee arthroplasty should be undertaken.

**Methods:**

A prospective online survey was done among members of EHS and EKA. The survey consisted of 27 questions. It includes basic information on demographics and details the participant’s agreement with each recommendation. The participant could choose among three options (agree, disagree, abstain). Recommendations focussed on pre-operative, peri-operative, and post-operative handling of patients and precautions.

**Results:**

A total of 681 arthroplasty surgeons participated in the survey, with 479 fully completing the survey. The participants were from 44 countries and 6 continents. Apart from adhering to National and Local Guidelines, the recommendations concerned how to make elective arthroplasty safe for patients and staff.

**Conclusion:**

The survey has shown good-to-excellent agreement of the participants with regards to the statements made in the recommendations for the safe return to elective arthroplasty following the first wave of the COVID-19 pandemic.

## Introduction

In recent months, the SARS-CoV-2 pandemic (COVID-19) has evolved rapidly in Europe, disrupting the personal, social, economic and professional lives of health professionals on a large scale. The overall goal of most governments in Europe has been to flatten the curve of infected SARS-CoV-2 patients and prevent a collapse of national health systems. The April 2020 SARS-CoV-2 survey completed by EHS and EKA members in Europe has confirmed the impact of SARS-CoV-2: this pandemic has resulted in a tremendous reduction in primary hip and knee arthroplasty procedures as shown in the survey. A broad consensus is needed about the factors that need to be in place before restarting such procedures.

Delaying hip and knee arthroplasty in patients with severe osteoarthritis (OA) may lead to increased opioid use. It is associated with lower clinical results and increased readmission rates after the index procedure. Moreover, when access to hip and knee arthroplasty is limited, as it is now in the wake of the COVID-19 sanitary measures, the direct and indirect costs for our health care and social systems are enormous. Many patients suffering from OA have to prolong their absence from work, request temporary unemployment benefits, and burden the public welfare system.

We are now entering a new phase in most European countries, where we can consider restarting elective hip and knee arthroplasty in a “post-pandemic” period. To date, the scientific basis for the existing guidelines is not robust; there is much room for an exchange of ideas between surgeons. The current concern is to map out the optimal trajectory for starting up elective hip and knee arthroplasties. As a result, the European Hip Society (EHS) and the European Knee Association (EKA) formed a panel of experts that have produced a consensus on how the safe re-introduction of elective arthroplasty should be undertaken. They have provided recommendations based on the available evidence [1]. This survey aimed to validate the recommendations by involving arthroplasty surgeons from a wide geographical area to promote then the recommendations for a safe return to elective joint arthroplasty across Europe and elsewhere.

## Materials and method

A prospective online survey was done online using SurveyMonkey (Portland, USA: https://www.surveymonkey.com) among members of European Hip Society (EHS) and European Knee Associates (EKA). A link to the survey was sent by email to all members of the EHS and the EKA and affiliated arthroplasty surgeons. The online survey was launched on 23rd May 2020 and concluded on 6th June 2020.

The survey consisted of 27 questions. It includes basic information on demographics and details the participant’s agreement with each recommendation. The participant could choose among three options (agree, disagree, abstain). The recommendations focus on three time periods; pre-operative, per-operative, and post-operative (Table 1).

**Table 1:** Questions and Statements from recommendations used in the survey

Questions	Statements
Resuming elective hip and knee arthroplasty in the setting of the SARS-CoV-2 pandemic	
1. When should elective hip and knee arthroplasty be resumed?	Elective hip and knee arthroplasty can be resumed when appropriate prerequisites concerning facilities, workforce, testing, supplies are met, and approval of local health authorities are obtained. Facilities in areas with low, or relatively low and stable incidence of SARS-CoV-2, can be allowed to provide care for patients needing non-emergent, non-SARS-CoV-2 healthcare
2. Are new triage/patient selection criteria needed for hip and knee arthroplasty patients once elective surgery is resumed?	Increased demand for hip and knee arthroplasty, coupled with limited hospital resources, will force surgeons to select which patients will receive hip and knee arthroplasties sooner than others. This will entail employing objective, transparent criteria in prioritizing patient selection to identify the patients most in need who also have lower risk factors for disease transmission and post-operative complications
3. What are the priority indications for elective adult hip and knee reconstruction?	There is universal agreement regarding the indications for urgent hip and knee arthroplasty procedures, such as femoral neck fractures, periprosthetic fractures, dislocations and acute infection, even in the setting of the SARS-CoV-2 pandemic. However, priority indications for non-emergency procedures in primary and revision hip and knee arthroplasty are lacking and should be clarified
4. What is the role of outpatient hip and knee arthroplasty/enhanced recovery protocols in light of the SARS-CoV-2 pandemic?	It is universally accepted that shorter hospital stays and treatment in a SARS-CoV-2-free environment decrease the risk of SARS-CoV-2 infection in patients undergoing elective surgery. Performing hip and knee arthroplasty with enhanced recovery protocols that decrease the length of stay in a facility or can be used in ambulatory care centres to allow for day surgery might be beneficial for reducing the risk of viral transmission. Transitioning to enhanced recovery systems requires full support, adoption of all the subspecialties involved and the possible approval of health care payers, which may not be possible in the pandemic setting
5. What is the impact of delaying hip and knee arthroplasty for the patients themselves and for society?	Delaying elective hip and knee arthroplasty has negative consequences on the quality of life of patients and negatively impacts society as a whole. The benefits of hip and knee arthroplasty should be carefully weighed against the risks of viral transmission and infection, complications and mortality in the mostly elderly population requiring joint arthroplasty
Resuming elective hip and knee arthroplasty in the setting of the SARS-CoV-2 pandemic: pre-operative phase	
1. What is the appropriate pre-operative clinical and laboratory screening and timeline for patients?	All patients undergoing surgery should have their temperature and oxygen saturation measured. A thorough medical history should be acquired and patients asked about any symptoms they may have, such as cough, fever, loss of smell or taste, headache or gastrointestinal disorders. Additionally, they should be questioned about recent travels and their occupation to stratify them into possible high-risk groups. Regarding the laboratory testing, it would be ideal for all patients to undergo RT-PCR testing for SARS-CoV-2 before the operation. However, local guidelines and the efficacy of tests must also be taken into consideration. If there is a paucity of available tests, then only high-risk patients should be tested. There is no indication for additional chest CT scans in the pre-operative screening. Time allowing, all surgical patients should apply social distancing principles for two full weeks prior to testing and the surgical procedure and self-quarantine in their home for the period between test acquisition and day of surgery
2. Is pre-operative tracking of patients, staff and relatives necessary?	All patients and staff will need to be screened for potential symptoms of SARS-CoV-2 prior to entering a hospital facility. In particular, staff must be routinely screened for potential symptoms. Isolation prior to surgery can be guided. All patients, staff and relatives, especially those with patient contact, need to be investigated for previous symptoms, travels abroad and possible contacts with populations at high-risk for SARS-CoV-2
3. Should spinal anaesthesia be routine?	Spinal anaesthesia can be considered safer for every negatively screened patient (with or without a SARS-CoV-2 test) than general anaesthesia, since the latter requires airway manipulation and endotracheal intubation, procedures that can more easily transmit SARS-CoV-2
– Should we only select patients as candidates who also consent to regional/spinal anaesthesia?	It cannot be made mandatory that patients consent to regional/spinal anaesthesia. Nevertheless, the benefits of regional anaesthesia should be thoroughly explained to the patients, and whenever possible, this should be strongly considered as the preferred means of anaesthesia
– If spinal anaesthesia does not occur, should we cancel the operation?	If general anaesthesia cannot be avoided, every precaution should be taken to avoid contamination. If a SARS-CoV-2-negative environment has been achieved that is as secure as possible, then general anaesthesia with informed consent can be considered
4. If the SARS-CoV-2 tests are positive, how long should the hip or knee arthroplasty surgery be postponed?	It is reasonable to assume that patients who test positive for SARS-CoV-2 should not be operated on and should be discharged from the hospital. These patients should remain in quarantine at least 14 days and until a subsequent test turns out negative (two confirmed negative PCR swab tests with a 24 h interval) and they are free of fever, cough or other symptoms
5. What kind of back-up plan is needed to guarantee quality care and patient safety in this pandemic situation?	The treatment of SARS-CoV-2 patients is placing a huge strain on the resources of many hospitals, to the detriment of treatment of other health problems. Safety of hip and knee arthroplasty patients remains of paramount importance. Complications of cardiac and respiratory origin are the most common problems needing acute care. As elective operations begin, every hospital should have ICU capacity available to accept and accommodate patients with cardiopulmonary or other significant complications. It should be assumed that, after the resumption of normal functions at hospitals and in society in general, there will still be a risk of another outbreak of the disease. In such a situation, hospitals should be prepared to return to a “safe mode” with reorganization of the wards and personnel. Every hospital should have in place a plan for emergency distribution of the personnel and wards before elective surgery resumes
Resuming elective hip and knee arthroplasty in the setting of the SARS-CoV-2 pandemic: perioperative phase 1. Is there a need for:	
– Modification or reorganization of hospital wards (patient density, bed density, medical and nursing staff density, etc.)?	In most orthopaedic departments, the workforces have been adjusted to accommodate fewer cases, due to the reduction in trauma cases and cancellation of elective procedures. As elective operations restart, the standard social distancing guidelines should be sufficient. Patient numbers in the clinical areas (wards, waiting areas, outpatient clinics) should be halved, with the patients spaced at least 2 m apart
– Separation of elective and trauma orthopaedic surgery (with regard to the general orthopaedic departments)?	SARS-CoV-2 screening practices should ensure the health and safety of patients and staff. SARS-CoV-2-negative elective hip and knee arthroplasty must be separated from the SARS-COV-2-positive trauma unit. Since all patients undergoing elective procedures are screened before administration and have tested negative for SARS-COV-2, they may be reluctant to go into certain settings for fear of viral transmission. There should be a physical separation of SARS-COV-2-positive and SARS-COV-2-negative patients and specific wards should be exclusively dedicated to treating patients with SARS-CoV-2 to reduce the risk of contaminating other patients
2. Should relatives/visitors or other supportive personnel be allowed onto the orthopaedic wards and operating suite?	Until normality returns, the number of visitors should be minimized as much as possible to reduce potential transmission of SARS-CoV-2 among patients, their family members and medical staff. This includes vendor representatives who travel to facilities to undergo, perform and support procedures. Limiting interactions between individuals and social distancing are part of the mainstream management against the spread of SARS-CoV-2. The number of visitors should be limited to those who are essential for a patient’s care. Although it is recognized that these rules create considerable anxiety for the patient, keeping all patients, relatives and staff safe from SARS-CoV-2 is the priority
3. Is there a need for extra disinfecting environmental procedures in between surgeries?	Standard protocols for cleaning and sterilizing instruments need to be meticulously applied Moreover, once the patient has departed from the operating room, this should be left empty for a specific period of time and all high-touch surfaces, including the anaesthetic machine and the anaesthetic work area, should be cleaned and disinfected with an Environmental Protection Agency (EPA)-approved hospital disinfectant. The length of time in between patients depends on the number of air exchanges per hour in the room or space in question
4. What role do ultra-clean operating theatres, orthopaedic exhaust suits and ultraviolet light systems play in both the wards/beds and the theatre?	Elective orthopaedic theatres and operative procedures, including personal protective equipment, are designed to reduce the risk of surgical site infection. There are no guidelines or protocols published for managing elective hip and knee arthroplasty procedures with respect to laminar flow in the presence of SARS-CoV-2. It is recommended that surgical helmets not be used as primary protection against aerosol and airborne disease. The surgeon should consider wearing an N95 mask as an added precaution when using a surgical helmet in patients considered to be SARS-CoV-2-negative in the pre-operative work-up Ultraviolet light has no evidence to support its routine use and poses a hazard to operating staff
5. What are the guidelines for personal protective equipment (PPE), in terms of availability and instructions regarding its use and what to wear in relation to the patient’s position in the perioperative chain?	During hip and knee arthroplasty, the use of power tools, burrs or electrocautery generates potentially infective aerosol. The major aim should be to avoid transmission of SARS-CoV-2 by aerosolization of blood or other body fluids. Hence, adequate personal protective equipment should be available and used during surgery. First and foremost, all patients undergoing elective surgery should wear a mask. Surgeons and the entire surgical team who scrub during procedures should ideally wear an exhaust suit, including a mask (preferably N95, filtering face piece [FFP2, or P3] and a face shield). In the absence of a face shield, protective eyewear may be used, but this is a compromise. In addition, scrubs should be frequently changed during the surgical day. Single-use gowns, single-use gloves and hair and shoe covers can also, theoretically, reduce the transmission of the virus
Resuming elective hip and knee arthroplasty in the setting of the SARS-CoV-2 pandemic: post-operative phase	
1. Since the impact on mind and body during joint arthroplasty is significant, our patients may have lower immunity and be more vulnerable to SARS-CoV-2 infection	As SARS-CoV-2-positive is an absolute contra-indication for elective surgery, the standard enhanced recovery protocol for reducing complications is imperative
2.Organization of a shelter-in-place post-operative period, home care, community nurses or informal care	Ideally, patients should be discharged home with the standard SARS-CoV-2 precautions being taken and only transferred to a nursing home in cases where that is not possible, since higher rates of SARS-CoV-2 may exist in those facilities
3. Avoid face-to-face contact for the orthopaedic follow-up and physical therapy guidance – Consider telemedicine	Web-based tools and telemedicine are preferred during this SARS-CoV-2 pandemic and may become the standard in the post-pandemic era
– Consider a new approach in wound closure technique in the setting of the SARS-CoV-2 pandemic	Post-operative in-office visits should be minimized, and digital health programmes allowing health care providers to closely monitor patients remotely should be supported. Self-administered wound management should become the standard of care for patients in the post-pandemic era
4. What is the advice for patients who develop SARS-CoV-2 symptoms?	In the event a patient develops SARS-CoV-2 symptoms or comes into contact with a SARS-CoV-2-positive person, they should seek health care advice. The patient should use the system in place in their country to seek the relevant advice. This may be through their primary care physician or an online or telephone advice service. Their orthopaedic surgeon and hospital should also be notified
5. Are special adjustments needed for post-op SARS-CoV-2 prophylaxis?	Since SARS-CoV-2-positive is an absolute contraindication or elective surgery, the standard enhanced f recovery protocol and SARS-CoV-2 preventive measures are imperative. Time allowing, all surgical patients should apply social distancing principles for the first two weeks after the operation and self-quarantine in their home

This survey did not require formal ethical approval with a practice dedicated to adult reconstruction.

## Results

**Fig. 1:**
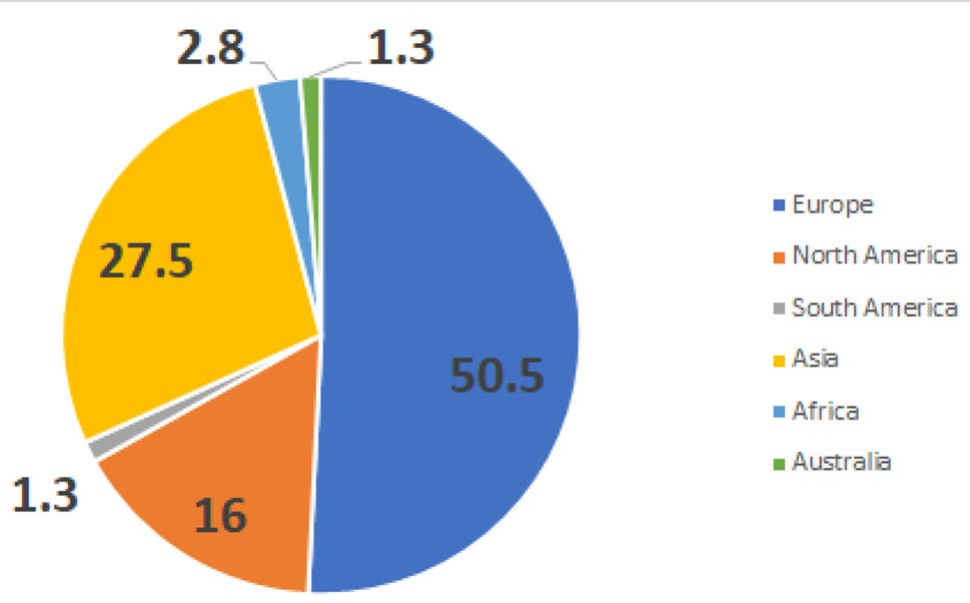
Geographical distribution of survey participants

A total of 681 arthroplasty surgeons participated in the survey, with 479 fully completing the survey. The geographical spread of this survey included surgeons from 44 different countries in 6 continents (Fig. 1). The EHS and EKA had a 22.1% and 20.9% response rate, respectively. The mean time in practice for all participants was 20 years (min 1 year–max 46 years).

The detailed results are shown in Table 2.

**Table 2:** Results of the survey

Time periods and questions	Agree *n* (%)	Disagree *n* (%)	Abstain *n* (%)	Response *n* (%)
Resuming elective hip and knee arthroplasty in the setting of the SARS-CoV-2 pandemic				
1. When should elective hip and knee arthroplasty be resumed?	653 (96)	18 (3)	10 (2)	681 (100)
2. Are new triage/patient selection criteria needed for hip and knee arthroplasty patients once elective surgery is resumes?	515 (82)	94 (15)	21 (3)	630 (93)
3. What are the priority indications for elective adult hip and knee reconstruction?	510 (83)	82 (13)	25 (4)	623 (92)
4. What is the role of outpatient hip and knee arthroplasty/enhanced recovery protocols in light of the SARS-CoV-2 pandemic?	450 (77)	76 (13)	59 (10)	585 (86)
5. What is the impact of delaying hip and knee arthroplasty for the patients themselves and for society?	530 (92)	27 (5)	19 (3)	576 (85)
Resuming elective hip and knee arthroplasty in the setting of the SARS-CoV-2 pandemic: preoperative phase				
1. What is the appropriate preoperative clinical and laboratory screening and timeline for patients?	435 (80)	80 (15)	31 (6)	546 (80)
2. Is preoperative tracking of patients, staff and relatives necessary?	379 (71)	121 (23)	36 (7)	536 (79)
3. Should spinal anaesthesia be routine?	420 (79)	74 (14)	40 (8)	534 (78)
– Should we only select patients as candidates who also consent to regional/spinal anaesthesia?	383 (72)	111 (21)	36 (7)	530 (78)
– If spinal anaesthesia does not occur, should we cancel the operation?	384 (73)	108 (20)	37 (7)	539 (78)
4. If the SARS-CoV-2 tests are positive, how long should the hip or knee arthroplasty surgery be postponed?	474 (90)	29 (6)	24 (5)	527 (77)
5. What kind of back-up plan is needed to guarantee quality care and patient safety in this pandemic situation?	476 (92)	19 (4)	24 (5)	519 (76)
Resuming elective hip and knee arthroplasty in the setting of the SARS-CoV-2 pandemic: perioperative phase				
1. Is there a need for				
– Modification or reorganization of hospital wards (patient density, bed density, medical and nursing stuff density, etc.)?	417 (81)	70 (14)	25 (5)	512 (75)
– Separation of elective and trauma orthopaedic surgery (with regard to the general orthopaedic departments)?	449 (89)	36 (7)	20 (4)	505 (74)
2. Should relatives/visitors or other supportive personnel be allowed onto the orthopaedic wards and operating suite?	458 (91)	32 (6)	13 (2)	503 (74)
3. Is there a need for extra disinfecting environmental procedures in between surgeries?	341 (69)	107 (22)	48 (10)	496 (73)
4. What role do ultra-clean operating theatres, orthopaedic exhaust suits and ultraviolet light systems play in both the wards/beds and the theatre?	320 (65)	96 (20)	76 (16)	492 (72)
5. What are the guidelines for personal protective equipment (PPE), in terms of availability and instructions regarding its use and what to wear in relation to the patient’s position in the perioperative chain?	359 (74)	83 (17)	4 (9)	446 (65)
Resuming elective hip and knee arthroplasty in the setting of the SARS-CoV-2 pandemic: post-operative phase				
1. Since the impact on mind and body during joint arthroplasty is significant, our patients may have lower immunity and be more vulnerable to SARS-CoV-2 infection	427 (88)	32 (7)	24 (5)	483 (71)
2.Organization of a shelter-in-place post-operative period, home care, community nurses or informal care	419 (87)	36 (8)	26 (5)	481 (71)
3. Avoid face-to-face contact for the orthopaedic follow-up and physical therapy guidance – Consider telemedicine	310 (65)	130 (27)	40 (8)	480 (70)
– Consider a new approach in wound closure technique in the setting of the SARS-CoV-2 pandemic	299 (62)	141 (29)	39 (8)	479 (70)
4. What is the advice for patients who develop SARS-CoV-2 symptoms?	455 (95)	11 (2)	13 (3)	479 (70)
5. Are special adjustments needed for post-op SARS-CoV-2 prophylaxis?	362 (76)	86 (18)	31 (7)	479 (70)

## Discussion

The survey has shown good-to-excellent agreement by the participants to the Statements made in the Recommendations for the safe return to elective arthroplasty following the COVID-19 pandemic. Although the response rate from both the EHS and EKA membership was low, at around 20%, it is notable that the mean time in elective arthroplasty of the participants was 20 years. This means that very experienced surgeons gave their opinions. Coupled with the global coverage of the survey, the mean time in elective arthroplasty is a proper validation for the recommendations.

## Conclusion

The survey has shown good-to-excellent agreement of the participants with regards to the statements made in the recommendations for the safe return to elective arthroplasty following the first wave of the COVID-19 pandemic.
